# Preparation and preliminary studies of porous fish collagen and chitosan materials enriched with microcapsules containing an active ingredient

**DOI:** 10.1038/s41598-025-95809-x

**Published:** 2025-04-03

**Authors:** Justyna Kozlowska, Agnieszka Ciesielska

**Affiliations:** 1 Faculty of Chemistry, Nicolaus Copernicus University in Torun, Gagarina 7, Toruń, 87-100 Poland; 2 Institute of Environmental Protection - National Research Institute, Slowicza 32, Warsaw, 02-170 Poland

**Keywords:** Fish collagen, Chitosan, Microcapsules, *Calendula officinalis* flower extract, Controlled release, Environmental sustainability, Drug discovery, Ecology, Ecology, Health care, Chemistry, Materials science

## Abstract

This study explores the development and characterization of advanced composite materials combining fish collagen and chitosan, enhanced with chitosan-based microcapsules encapsulating an active ingredient—*Calendula officinalis* flower extract—hrough ionic gelation using various surfactants (Span 80, Tween 80 and Span80/Tween 80). Collagen was successfully extracted from northern pike scales and integrated with chitosan to create porous, three-dimensional matrices by the lyophilization process. Various amounts of microcapsules were incorporated into the matrices, and the structure of the obtained materials, their mechanical properties, swelling capacity, and susceptibility to degradation were assessed. Matrices with microcapsules exhibited high porosity, substantial swelling capacity, and improved mechanical properties compared to matrices without them. Microcapsules enabled the controlled release of active ingredients, demonstrating potential applications cosmetic industry. This research aligns with current trends in the cosmetics industry, such as the use of sustainable and eco-friendly materials derived from renewable resources like fish waste, the emphasis on natural and bioactive ingredients such as plant extracts, and the development of advanced delivery systems for controlled release of active compounds. The study addresses consumer demand for biodegradable and non-toxic materials, reducing environmental impact while enhancing product efficacy and safety.

## Introduction

Due to economic growth and the subsequent variety of diets, global fish production has surged rapidly. Fish production was projected to reach 194 million tons by the close of 2026^1^. Consequently, the amount of fish waste generated has significantly increased, posing a severe environmental threat. This situation has accelerated the need for effective treatment and proper disposal of fish waste. Annually, over 28 million tons of by-products, such as fish heads, viscera, backbones, scales, and skin, are produced^2^. Meanwhile, fish waste is a plentiful source of various valuable components, such as proteins, carbohydrates, polyunsaturated fatty acids, and other minerals. Therefore, by-products from fish processing can be used in animal feed, food production, and other industries. Despite this potential, these by-products are often discarded, leading to organic pollution^3^.

One of the most valuable ingredients found in fish waste is collagen. Collagen is a biopolymer found in the extracellular matrix, comprising 25–30% of animal protein content. It is among the most prevalent proteins in the animal kingdom^4^. Collagen is easily accessible and has numerous valuable properties, including biocompatibility, biodegradability, weak antigenicity, antimicrobial activity, and non-toxicity^5^. Therefore, this protein is considered one of the most significant and versatile biopolymers in biomaterials research. Among the different types of collagen, type I collagen is widely utilized as a biomaterial in biomedical applications, including tissue engineering constructs and wound dressing systems^6^. The industrial use of type I collagen is growing not only in the biomedical industry but also across sectors such as cosmetics, pharmaceuticals, and food^7^. Type I collagen is typically extracted from animal tissues such as bovine or porcine skin and bovine or equine Achilles tendons. However, collagen from these animal sources presents potential viral and prion contamination risks. The outbreaks of bovine spongiform encephalopathy (BSE) and transmissible spongiform encephalopathy (TSE) in pigs and cattle have led to restrictions on using collagen products derived from these sources^8^. Currently, due to risks associated with the use of animal collagen as well as environmental damage caused by waste disposal of fish processing, researchers are interested in developing strategies to extract collagen from fish waste material from various fish species for practical uses^9^.

Numerous studies have examined collagen’s characteristics and potential isolation methods from multiple fish species. Both freshwater and marine fish have been extensively researched. In recent years, collagen has been isolated from several tissues of different fish species, including Pacific saury (*Cololabis saira*)^10^, Amur sturgeon (*Acipenser schrenckii*)^11^, *Catla catla* and *Cirrhinus mrigala*^12^, Japanese sea bass (*Lateolabrax japonicus*) and Nile tilapia (*Oreochromis niloticus*)^13^, Blue Shark (*Prionace glauca*)^14^. Despite the numerous benefits of fish collagen, it also has several drawbacks, including a low denaturation temperature, poor mechanical properties, and a high degradation rate. Nevertheless, considerable efforts have been made to enhance its mechanical properties and degradation profile through methods such as chemical^15^, physical^16^, or enzymatic cross-linking^17^, and blending with other polymers^18^.

In addition to waste from fish processing, substantial marine residues are obtained from crustacean exoskeletons and mollusk shells. These materials are also utilized for the extraction of biopolymers like chitin^19^. Chitin is a naturally occurring biopolymer found in the exoskeletons of crabs, shrimp, and insects, as well as in the cell walls of fungi and algae. Its utility is significantly enhanced when it is converted into chitosan through a partial deacetylation process performed under alkaline conditions^20^. Chitosan is a quite unique polysaccharide notable for having primary amines along its backbone. This structural feature grants the polymer valuable physicochemical properties and enables specific interactions with proteins, cells, and living organisms^21^. When protonated in acidic conditions, this polycation can be combined with various anionic polymers to create materials in diverse forms, including films, sponges, porous membranes, hydrogels, and nanofibers^22^. Over the past ten years, interest in chitosan-based materials has surged due to their biocompatibility, biodegradability, non-toxicity, and non-allergenic characteristics. Thanks to its antimicrobial and anti-inflammatory properties, chitosan has demonstrated significant potential as a biomaterial for wound healing applications^23^.

The diverse properties of fish collagen and chitosan, derived from marine by-products and processed materials, suggest a promising future for these biopolymers as biomaterials, particularly in the field of wound dressing. Numerous studies have highlighted their potential, with significant literature available on collagen and modified collagen, including blends with chitosan, for wound healing and other topical applications^24–26^.

In our research, we decided to obtain a prototype of a three-dimensional porous material based on a mixture of fish collagen and chitosan intended for the potential application to the skin. For this purpose, we used collagen isolated in laboratory conditions from northern pike (*Esox lucius*) scales^27^. Moreover, to increase the effectiveness of the matrix, we decided to enrich it by adding microcapsules with the active ingredient. We used the *Calendula officinalis* flower extract as a model active substance. The materials obtained may be potential applied in cosmetic industry. The cosmetics industry faces increasing scrutiny for its environmental footprint, driven by the high demand for products and the extensive use of non-sustainable resources. This study aims to address these challenges by leveraging renewable biopolymers such as fish collagen and chitosan, combined with advanced encapsulation techniques, to develop innovative and eco-friendly materials. By integrating sustainable raw materials and controlled-release systems, this research provides a promising pathway toward reducing environmental pollution while meeting consumer expectations for effective, biodegradable, and safe cosmetic products. Such an approach represents a meaningful step forward in aligning the cosmetics industry with global sustainability goals^28,29^.

## Materials and methods

### Materials

Collagen type I (COL) from scales of freshwater fish - northern pike (*Esox lucius*) was obtained and characterized in our laboratory^27^. In brief, fish scales were treated with NaOH to remove non-collagenous proteins, followed by EDTA. Collagen was extracted using 0.5 M acetic acid and then separated by a salting-out process, where NaCl was added to a final concentration of 2.5 M to precipitate the collagen. The precipitate was collected by centrifugation (13,000 × g, 30 min), redissolved in acetic acid, and dialyzed to remove excess salt. Finally, the purified collagen was freeze-dried.

Chitosan (CTS), 1-ethyl-3-(3-dimethylaminopropyl) carbodiimide (EDC), N-hydroxysuccinimide (NHS), pentasodium triphosphate (TPP), Tween 80, Span 80, Folin-Ciocalteu reagent, and gallic acid were obtained from Sigma-Aldrich (Poznan, Poland). The hydro-glycolic (propylene glycol/water 80:20) extract of pot marigold (*Calendula officinalis*) flowers was procured from Provital S.A. (Barcelona, Spain). All other reagents were sourced from Avantor Performance Materials Poland S.A. (Gliwice, Poland). All chemicals used were of analytical grade.

### Microencapsulation procedures

The chitosan-based microcapsules were prepared using an ionic gelation method as described by Sukmawati et al., with modifications^30^. Firstly, a 1% chitosan solution in 1% acetic acid, containing 0.1% pot marigold flower extract was prepared. Subsequently, one of the surfactants—Span 80, Tween 80, or a 1:1 weight ratio mixture of Tween 80 and Span 80—was added dropwise under continuous stirring at approximately 400 rpm for 2 h to achieve a homogeneous solution, ensuring a final surfactant concentration of 0.5%.

Then, TPP solution (3 mg/ml) was gradually introduced dropwise into the resulting surfactant under constant stirring. This mixture was homogenized at 2000 rpm for 10 min, followed by 24 h of mixing with a magnetic stirrer.

### Matrices preparation

0.6% chitosan solution in 0.2 M acetic acid and 0.6% fish collagen solution were prepared. A mixture of chitosan and collagen solutions was prepared in a weight ratio of 1:1. Then, microparticles were added to the resulting solution in appropriate amounts to obtain mixtures with weight ratios of polymers (collagen and chitosan) to microparticles of 1:2 and 1:4. These mixtures were subsequently frozen (− 20 °C) and lyophilized (− 55 °C, 5 Pa, 48 h) using a CHRIST ALPHA 1–2 LD plus lyophilizer (Martin Christ, Osterode am Harz, Germany).

The collagen/chitosan matrices obtained by freeze-drying were cross-linked using an EDC/NHS cross-linking mixture. The samples were chemically modified with 50 mM EDC and 25 mM NHS, dissolved in 98% ethanol at room temperature for 4 h. Following this, the samples were washed twice with 0.1 M Na_2_HPO_4_ over a period of 2 h. To remove any remaining cross-linking compounds, the samples were washed four times with distilled water for 2 h, with the water being changed every 30 min. Finally, the cross-linked samples were frozen (− 20 °C) and freeze-dried for 48 h (− 55 °C, 5 Pa). The collagen/chitosan matrices containing microcapsules obtained using Span 80, Tween 80, and Tween80/Span80 were named COL/CTS/S/1:2 and COL/CTS/S/1:4, COL/CTS/T/1:2 and COL/CTS/T/1:4, COL/CTS/S/T/1:2 and COL/CTS/S/T/1:4, respectively, where 1:2 and 1:4 represent the weight ratio of polymers to microcapsules in a given matrix. The matrix without microspheres was a control sample named COL/CTS.

### Characterization of microcapsules

#### Structure and size

The microcapsules obtained by the ionic gelation method were observed by the optical microscope Motic SMZ-171 BLED (Hong Kong, China) at magnification x20, and the cross-section of microcapsules was observed using microscope Delta Optical (Nowe Osiny, Poland).

#### In vitro release profile

The microcapsules were weighed in triplicate and placed into 24-well polystyrene plates. Subsequently, 2 ml of acetate buffer (pH = 5.4), chosen to simulate the natural conditions of human skin, was added to each well. The plates were incubated at 37 °C. Samples were collected at intervals of 30 min, 1 h, 2 h, 3 h, 24 h, 72 h, 96 h, 120 h, and 144 h, with fresh acetate buffer pre-warmed to 37 °C being added to the microcapsules after each collection. The collected samples were then frozen at – 20 °C. Once all samples were collected, the total phenolic compound content was determined using the Folin-Ciocalteu assay^31^. The absorbance was measured at 725 nm with a UV-Vis spectrophotometer (UV-1800, Shimadzu, Kyoto, Japan).

### Characterization of materials

#### Microstructure

The structure of the obtained three-dimensional materials was analyzed using scanning electron microscopy (SEM) with the Quanta 3D FEG scanning electron microscope from Quorum Technologies (Lewes, UK). Before analysis, the sample surfaces were coated with a thin layer of gold and palladium.

#### Porosity measurements

The porosity (Є) of the obtained materials was measured using the liquid displacement method^32^. Isopropanol was used as the displacement liquid, which does not dissolve the matrix-forming polymers. The matrix sample was weighed (W) and placed in a graduated cylinder containing 3 ml of isopropanol (V_1_). After 5 min, the new liquid level (V_2_) was recorded. The sample was then carefully removed, and the remaining volume of isopropanol (V_3_) was noted. This procedure was repeated in triplicate for all matrix types. The porosity (Є) of the matrices (1) was calculated using the following formulas:

1 $$\upepsilon = ({V_1} - {V_3}) / ({V_2} - {V_3}) \cdot 100\%$$

#### Swelling properties

A piece of each dry matrix was weighed (W_d_) and then immersed in 5 ml of phosphate-buffered saline (PBS, pH = 5.7), selected to simulate the natural conditions of human skin, for intervals of 15 min, 30 min, 1 h, 2 h, and 3 h at room temperature (22 °C). After each specified period, the samples were removed from the PBS solution and weighed again (W_w_). This test was conducted in triplicate for all matrix types. The swelling ratio of the matrices (2) was determined as the ratio of the increase in weight to the initial weight, calculated as follows^33^:

2 $$swelling{\text{ }}ratio{\text{ }}={\text{ }}\left( {{W_w} - {W_d}} \right)/{W_d}\cdot100\%$$

#### Mechanical properties

The mechanical properties were evaluated using a mechanical testing machine (Z.05, Zwick/Roell, Ulm, Germany). Before testing, the cylindrical samples were measured for diameter and height. The tests were performed at a compression speed of 50 mm/min. Young’s modulus was determined from the slope of the stress-strain curve within the linear region (strain between 0.05% and 0.25%). The results were recorded using the testXpert II software. The values presented are the averages calculated from five measurements for each matrix type.

#### Degradation measurements

Dry samples (W_b_) were weighed, placed in 12-well polystyrene plates, and immersed in 5 ml of PBS (pH = 5.7), chosen to simulate the natural conditions of human skin. The samples were incubated at room temperature for 1, 2, 3, 7, 14, 21, and 28 days. After each period, the samples were removed from the PBS buffer, rinsed three times with deionized water, frozen, lyophilized, and weighed (W_a_). The materials were subjected to degradation measurements in triplicate. The percentage weight loss (3) was calculated according to the following equation^34^:

3 $$weight{\text{ }}loss{\text{ }}={\text{ }}\left( {{W_b} - {W_a}} \right)/{W_b}\cdot100\%$$

### Statistical analysis

Statistical analysis of the data was performed using commercial software (SigmaPlot 14.0, Systat Software, San Jose, CA, USA). All of the results were presented as a mean ± standard deviation (SD) and were statistically analyzed using one-way analysis of variance (one-way ANOVA). Multiple comparisons versus the control group between means were performed using the Bonferroni t-test with the statistical significance set at *p* < 0.05.

## Results and discussion

### Microcapsules

**Fig. 1:**
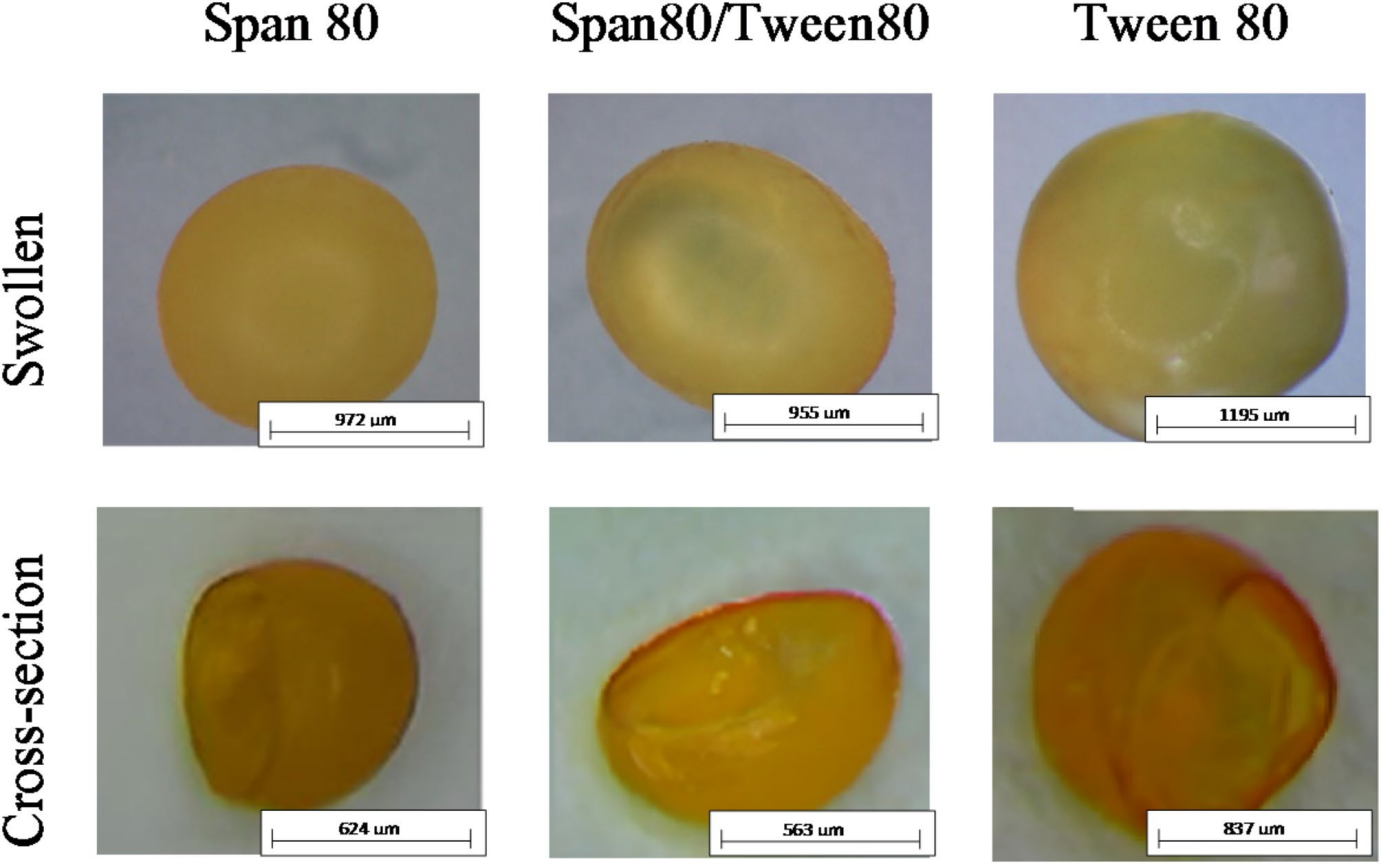
Microscope images of swollen microcapsules prepared from chitosan and *Calendula officinalis* flower extract by ionic gelation method using different surfactants.

Figure 1 presents images of the obtained microcapsules. The microcapsules produced by ionic gelation methods are shown to be swollen. Slight variations can be observed between the microcapsules obtained with different surfactants. Morphological observations indicated that the microcapsules were either spherical or oval. The swollen microparticles had a regular, nearly spherical shape and smooth surface. Cross-sectional analysis confirmed the successful formation of capsules, revealing their characteristic wall and core structure, which is typical for encapsulation systems.

The diameters of all the obtained microcapsules are presented in Table 1. Microcapsule diameters were measured using a Motic SMZ-171 BLED stereoscopic microscope. The largest sizes were found in the case of swollen capsules obtained using Tween 80, with diameters averaging about 1205 ± 10.3 μm, whereas after dried, they were smaller, approximately 836 ± 10.8 μm. Due to their diameter exceeding 1000 μm, these swollen particles are classified as macrocapsules rather than microcapsules^35^. Furthermore, the microcapsules created using the ionic gelation method with Span 80 or a mixture of Span 80 and Tween 80 had similar sizes, around 967–976 μm. The diameters of the dry microcapsules produced using Span 80 and the Span 80/Tween 80 mixture were 622 μm and 561 μm, respectively. This indicates that the type of surfactant used in the encapsulation process significantly affected their sizes and slightly impacted their shape. Similar conclusions were reached by Li et al. as a result of research on obtaining microcapsules using various surfactants (Tween 20, Tween 40, Tween 60, Tween 20/Span 80 at a 1:1 ratio, Tween 20/SDBS at a 1:1 ratio, and Span 80) through an emulsion-ionic gelation method^36^. After encapsulating citrus essential oil in chitosan microcapsules using six different emulsifiers, microcapsules with varying sizes and shapes were obtained, despite the fact that both Tween 80 and Span 80 are nonionic emulsifiers.

**Table 1:** Diameters of microcapsules obtained by ionic gelation method using different surfactants.

Type of surfactant	Size of microcapsules [µm]	
	Swollen	Dry
Span 80	976 ± 7.6	622 ± 8.3
Span80/Tween80	967 ± 13.3	561 ± 9.5
Tween 80	1205 ± 10.3	836 ± 10.8

**Fig. 2:**
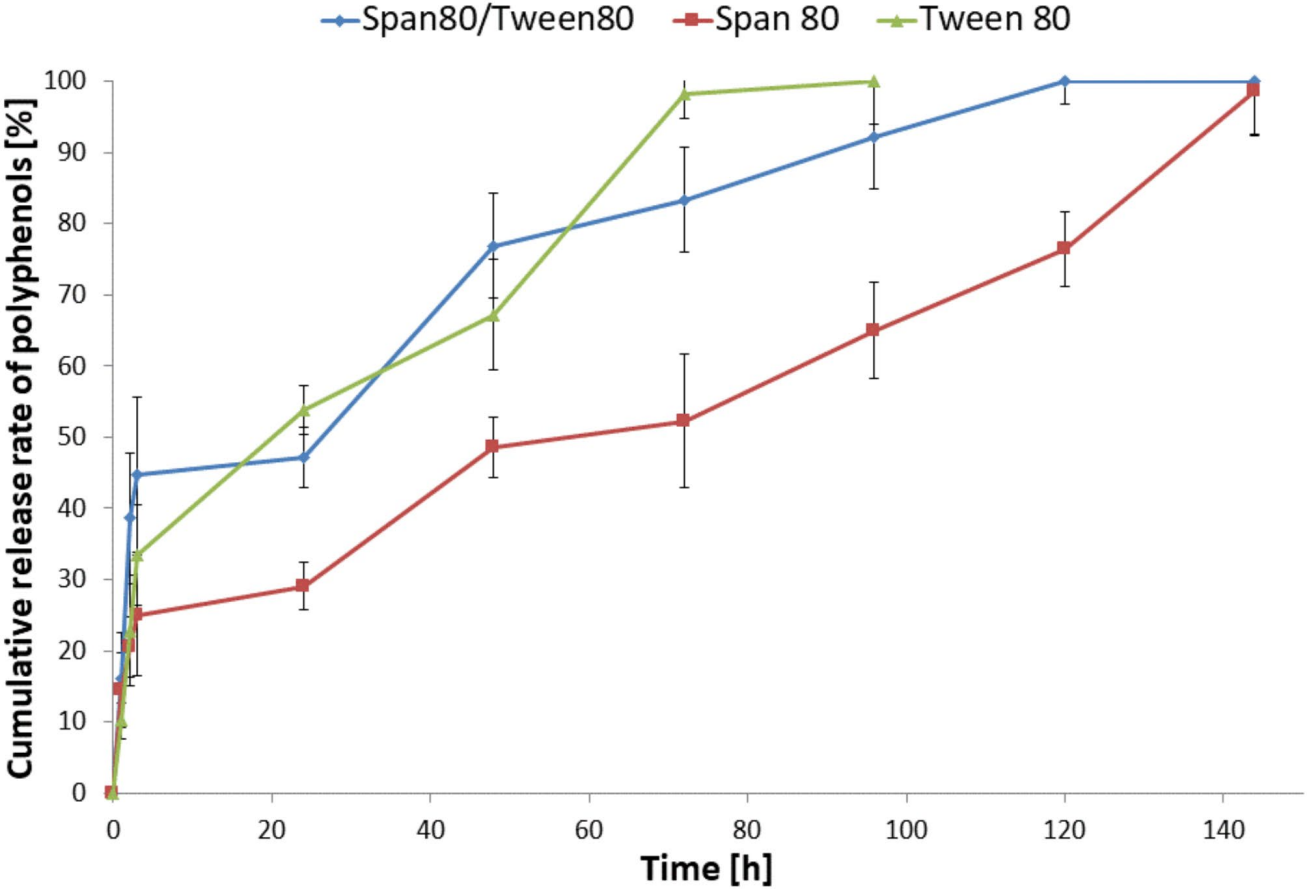
In vitro release assays of microcapsules based on chitosan obtained by ionic gelation method using different surfactants (*n* = 3, data are expressed as the mean ± SD).

The pot marigold flower extract release profile embedded in the microcapsules based on chitosan is shown in Fig. 2. The results indicate that the type of surfactant used during encapsulation affects the rate of active substance release. As one can see, the active substance encapsulated in the chitosan microcapsules made using Tween 80 was released entirely after 96 min of incubation in an acetate buffer at 37 °C. On the other hand, encapsulated marigold flower extract was released only after 120 and 144 h from microcapsules obtained using the Tween80/Span80 and Span 80, respectively.

In addition, we can observe a more intensive release of marigold flower extract during the first 3 h of the test − 25%, 45%, and 33% plant extract were then released from capsules made using Span 80, Span 80/Tween 80 and Tween 80, respectively. A so-called burst release of Calendula officinalis flower extract characterized the first release stage. Because of their relatively low molecular weight, polyphenols can readily diffuse through the polymer matrix^37^. Surface diffusion and the burst effect are observed, especially with smaller sizes of the encapsulated particles^38^. After this time, the active substance is released less rapidly. This may have been caused by the release of plant extract from micro-particles by gradual diffusion of polyphenols through the chitosan membrane. The results confirm that surfactant types affect the release rate of microcapsules’ active substances. Li and Zhong reported that surfactants can delay the release of active substances into water due to their partially hydrophobic nature^39^. They investigated the effects of Span 80, Tween 20, and their mixture on the properties of kudzu starch membranes containing ascorbic acid. Each surfactant reduced the surface tension of the film-forming solution and delayed the release of ascorbic acid from the membrane into the water.

Even though the microcapsules obtained using Tween 80 were bigger than others, the extract from pot marigold flowers was released the fastest. These microparticles had the thinnest polymer shell, which can be seen in the photo of the cross-section of the microcapsule, on which the shell is visible (Fig. 1). On the other hand, the microcapsules obtained using the Tween80/Span80 and Span 80 were smaller, but had a thicker polymer shell, so the active substance was released longer. Therefore, microcapsules with a thicker polymer shell are subject to slow degradation and remove the active substance more slowly than microcapsules with a thinner polymer shell. Microcapsules with different relative shell thicknesses show different release rates^40^.

### Matrices with microcapsules

**Fig. 3:**
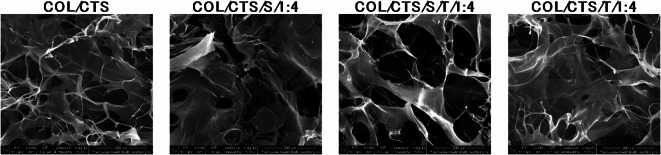
Scanning electron microscopy (SEM) images of COL/CS matrices with and without microparticles in magnification 500x (scale bar = 200 μm).

Figure 3 presents images of the produced 3D matrices obtained by scanning electron microscopy (SEM). Freeze-drying of polymer solutions led to the formation of three-dimensional matrices due to the sublimation of ice crystals formed during the freezing step. When a material is frozen, the solvent solidifies into ice crystals. During the lyophilization process, these ice crystals are removed directly through sublimation under low pressure and temperature, leaving behind voids or pores in the structure where the ice crystals were originally located^41^.

The morphology of the created COL/CTS matrices featured irregular macropores and outstanding interconnectivity. The size of the capsules rendered them unobservable with SEM.

**Table 2:** Porosity (Є) (*n* = 3), Young’s modulus values (E_mod_) (*n* = 5) and swelling ratio (*n* = 3) of COL/CTS porous matrices containing microcapsules based on Chitosan (data are expressed as the mean ± SD, * significantly different from the control—OL/CS (*p* < 0.05).

Sample	Є (%)	E_mod_ [kPa]	Swelling ratio [%]
COL/CTS	86.6 ± 1.3	8.15 ± 1.74	3754 ± 416
COL/CTS/S/1:2	84.1 ± 1.4	7.42 ± 1.23	3677 ± 493
COL/CTS/S/1:4	84.7 ± 2.4	9.77 ± 0.99	2715 ± 656
COL/CTS/S/T/1:2	85.2 ± 3.2	10.4 ± 1.24	3321 ± 682
COL/CTS/S/T/1:4	84.7 ± 2.4	10.49 ± 1.44*	3585 ± 785
COL/CTS/T/1:2	85.5 ± 2.1	9.34 ± 0.28	3675 ± 911
COL/CTS/T/1:4	84.5 ± 1.7	10.7 ± 1.41*	3250 ± 539

All samples are porous, with porosity exceeding 80% (Table 2). The sample without microcapsules has the highest porosity at 86.6%, while adding microparticles slightly reduces the porosity of the samples. It can be seen that samples with a lower ratio of microcapsules to polymers in the matrix (1:2) exhibit higher porosity than those with a higher ratio of microparticles (1:4), likely due to the increased density and reduced void spaces caused by the incorporation of higher amounts of solid microparticles, which partially fill the pores formed during the freeze-drying process^42^.

Samples with a lower concentration of microparticles demonstrated lower Young’s modulus values compared to those with a higher concentration of microparticles (Table 2). The increase in Young’s modulus corresponds to reduced deformation under applied stress, indicating greater stiffness in materials with higher microparticle content. This observation confirms that the addition of microcapsules to the matrix enhances its mechanical properties, likely due to the reinforcement effect of the microcapsules. Their presence increases the matrix density and mechanical strength while simultaneously reducing its flexibility. This effect was most pronounced in the samples COL/CTS/S/T/1:4 and COL/CTS/T/1:4, which exhibited the highest Young’s modulus values.

All tested materials exhibited excellent swelling behavior in aqueous conditions (Table 2). The swelling capacity of all samples obtained is very high and increases up to 2 h, after which it stabilizes. The maximum degree of swelling was recorded after 2 h of incubation in the buffer (Table 2), and after that time, it remained relatively constant (results not shown). The highest swelling (3754 ± 416%) was observed in the control sample COL/CTS without containing microcapsules. The addition of microcapsules decreased the swelling ability of the materials. It can therefore be seen that the more microparticles, the lower the swelling capacity of the sample; for example, the swelling ratio is 3677 ± 493% and 2715 ± 656% for COL/CTS/S/1:2 and COL/CTS/S/1:4, respectively. It can, therefore, be seen that the more microparticles are incorporated into the matrix, the lower the swelling capacity of the sample, likely due to the reduced availability of polymer chains for water binding, the physical blocking of pore spaces by the microparticles, and the increased density and rigidity of the material^43^.

**Fig. 4:**
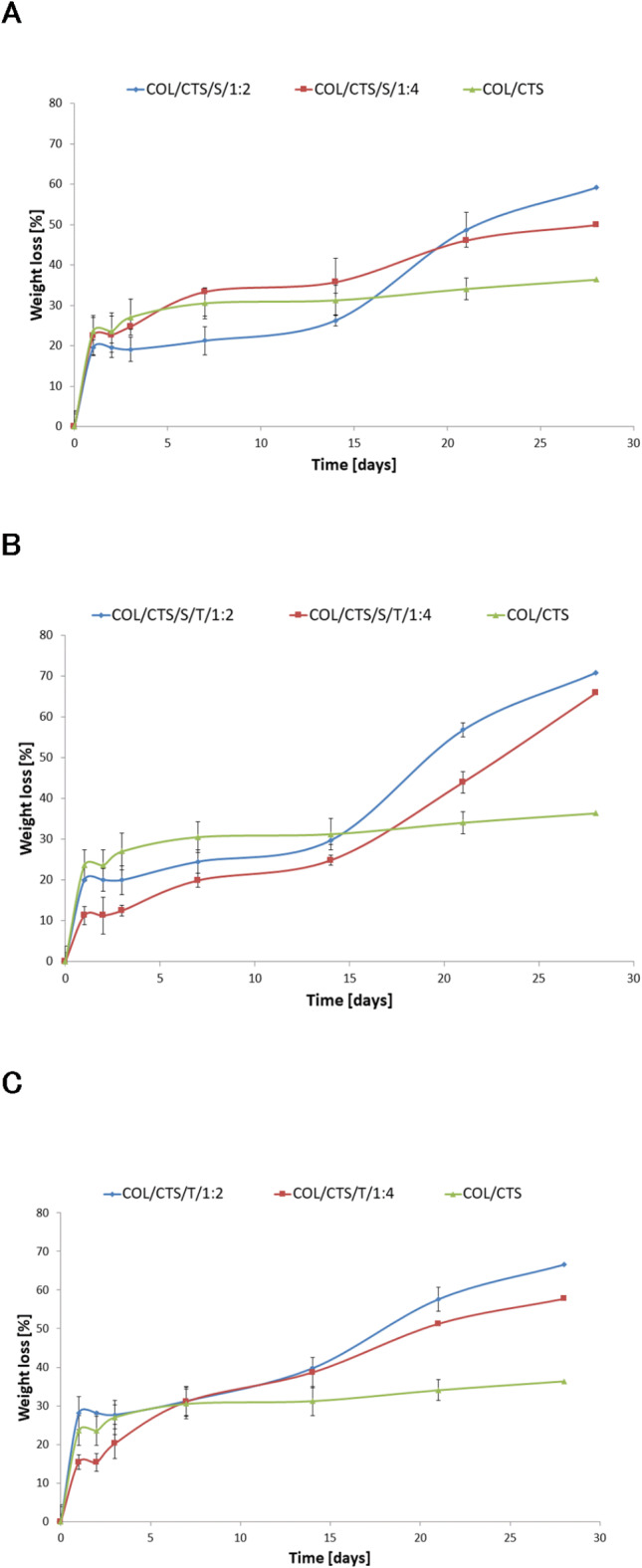
The values of weight loss during the degradation of COL/CTS—based matrices with incorporated different microcapsules: (A) matrices with capsules obtained using Span 80; (B) matrices with capsules obtained using Span 80/Tween 80; (C) matrices with capsules obtained using Tween 80; COL/CTS—matrix without microcapsules; (*n* = 3, data are expressed as the mean ± SD)

The consideration of material degradation is crucial as it indicates the shelf life of materials. The study was conducted for four weeks, representing long-term degradation (Fig. 4). During the initial three days, significant weight loss was observed in each type of material. After this period, the weight of all matrices decreased gradually up to 2 weeks. It was found that incorporating microparticles into the matrices significantly alters their susceptibility to degradation. In the case of matrices with microcapsules, we observed increased weight loss of the material, indicating reduced material stability. Interestingly, after 14 days of testing, we can observe again an increase in the intensity of the degradation process in the case of the matrices containing microcapsules. The mass losses of these materials are getting more significant. Samples containing more microcapsules (1:4 to polymer ratio) degraded slower than samples containing fewer microcapsules (1:2). We observed a similar result in our previous studies, assessing the degradation of matrices enriched with microparticles. It is plausible that the increased weight loss in the polymer matrices containing microparticles may have been due to their leaching out from the samples during the degradation process^44^.

In this study, porous three-dimensional collagen/chitosan matrices incorporating chitosan-based microcapsules were successfully developed and analyzed. These materials exhibited high porosity and significant swelling capacity. The incorporation of microcapsules resulted in improved mechanical properties compared to matrices without microcapsules, albeit with a slight reduction in swelling capacity and porosity.

This research led to the creation of an eco-friendly lyophilized material derived from fish waste, aligning with current trends in the cosmetics industry. The collagen/chitosan matrices enriched with microcapsules offer a sustainable alternative to traditional cosmetic formulations. A key advantage of this material is its water-free form, which eliminates the need for preservatives. This feature directly addresses consumer demand for natural, safe, and environmentally friendly products.

The lyophilized material can be rehydrated immediately prior to use, facilitating the preparation of fresh formulations. Upon rehydration, the encapsulated active ingredient, securely retained within the microcapsules, is released in a controlled manner. This ensures the preservation of its biological activity and efficacy during application, providing a novel approach to the delivery of bioactive compounds while maintaining their stability and functionality.

To fully validate the proposed benefits of this system, further studies are required. Future research should focus on assessing the long-term stability of encapsulated active ingredients, their release kinetics under real-world conditions, and their biological efficacy. These investigations will be essential for optimizing the material properties and realizing its full potential as a sustainable solution for controlled-release systems in cosmetic formulations.

## Conclusions

This study demonstrated the successful development of composite materials based on fish collagen and chitosan, enriched with chitosan-based microcapsules encapsulating *Calendula officinalis* extract. The obtained materials exhibited high porosity, improved mechanical properties, and controlled release profiles, underscoring their potential for applications in the cosmetics industry. This research contributes to the field of cosmetic science by offering an innovative approach to utilizing renewable biopolymers derived from fish waste, promoting sustainability while addressing the demand for biodegradable and eco-friendly materials. The incorporation of microcapsules provides a foundation for advanced delivery systems that enhance the efficacy of cosmetic formulations. Future studies will focus on application-oriented research to further explore the functionality of the developed materials. This will include assessing their antibacterial and antioxidant properties, and encapsulation efficiency in practical conditions. These investigations will provide valuable insights into their broader potential in sustainable cosmetic applications.

## Data Availability

All data generated during this study are included in this published article.
